# An Application of a Magnetic Impulse for the Bending of Metal Sheet Specimens

**DOI:** 10.3390/ma15103558

**Published:** 2022-05-16

**Authors:** Ján Moravec, Miroslav Blatnický, Ján Dižo

**Affiliations:** Faculty of Mechanical Engineering, University of Žilina, Univerzitná 8215/1, 010 26 Žilina, Slovakia; jan.moravec@fstroj.uniza.sk (J.M.); miroslav.blatnicky@fstroj.uniza.sk (M.B.)

**Keywords:** bending process, magnetic impulse, metal sheet, punch

## Abstract

Currently, classical methods for the creation of various shapes and bending angles of metal sheet parts are applied. They are represented by the so-called all-metal forming tools. Non-standard methods, which in some cases exceed conventional technical solutions, are used in the practice to a minor extent. This is an area of interest from the point of view of ecology, because the shaping process performed in this way does not burden the environment in any considerable way. The knowledge presented in this work is obtained based on experiments in laboratory conditions. The list of literature contains mainly works from the recent period. The research represents a contribution to the great mosaic of magnetism. The aim of the current paper is to also verify the possibilities of the suitability of a special tool in the formation of metal sheet specimens using the application of the so-called forming with a free core. Additional benefits of the experimental work and their results are anticipated. The contribution is complemented by detailed calculations and diagrams. The practical contribution and research is that the device used for forming has been successfully tested. It turns out that the presented method is suitable for further development. The method has proven that is it suitable for industrial applications where simple shapes are produced.

## 1. Introduction

For decades, forming technology has occupied a leading position in the field of mechanical engineering. Its main advantage is the significant savings of materials, which are proving to be very suitable and appropriate in the current pressure for green production (in general and worldwide). Another advantage of forming is its relatively easy process of automatization. The significant importance of forming technology is also the fact that it allows a substantial increase in labor productivity. This is preferably reflected in a shortening of the production cycle and a reduction in labor. However, the introduction of new knowledge into the production and use of the advantages of forming technologies also depends on a proper design for forming tools. The main principle consists of the application of new procedures by using physical knowledge, which is a large reservoir of stimuli. Here, forming mainly takes place in the so-called non-rigid instruments. They are forming tools, whose design and production take into account the use of new solutions without the need for all-metal tools. Nothing can be underestimated when applying forming processes. The very way of the presented sphere belongs to the spectrum of unconventional technological processes of forming. The research presented in [1] highlights the investigation of the influence of coil length on electromagnetic forming. Van Hunsel et al. [2] studied the possibilities of creating of shaped fittings in a special tool of their own design. Meng et al. researched the effects of important parameters during the electromagnetic forming of sheets made of a magnesium alloy [3]. The contribution by Golovaschenko [4] is important form the point of view of practical possibilities and applications of electromagnetic forming for the automotive industry. Unger et al. [5] contributed with their research on the overview of the modeling of forming process of electromagnetic metal, where many research activities are needed. Woodward et al. [6] developed knowledge about the production by means of non-conventional technologies of forming. This article focuses on the production of components for the aviation industry. The two-stage process of electromagnetic forming of materials is described in detail in [7]. Okoye et al. [8] dealt with the problem of the application of electromagnetic-assisted stamping on a particular example of the gradual pressing of metal sheets. The gradual pressing of metal sheets is one of the most important processes in this area. The text of the contribution presented by Krausel et al. [9] offers a detailed overview of the problem of dividing metal sheets with an application of pulse electromagnetic fields. The research [10] describes in detail how electromagnetic forming is applied to the production of components for the automotive industry. The contribution prepared by Bellito et al. [11] supplemented the described phenomenon of electromagnetic forming. Without the detailed knowledge of crystalline structures and magnetism, it would be difficult to understand the deep and mutual bounds of electromagnetism and the magnetism of materials. The introduced overview of the current research can be considered as sufficient and authors have been discovered during the research. It is also obvious that electromagnetic forming is one of the advanced and ecological technologies for metal forming and a great future awaits it in this century.

Nowadays, for the creation of various shapes and bending angles of sheet metal parts, classical methods are applied in practice, presented by the so-called all-metal forming tools. It is necessary to use a single-purpose tool for each part and bend, respectively, another method of mechanical bending. To a minor extent, non-traditional methods are used in practice, which, in some cases, exceed conventional technical solutions. This phenomenon is also caused by the financial capabilities of manufacturing companies [12,13,14,15,16]. In the presented analysis in manufacturing companies, we came across this phenomenon, i.e., a lack of funds. This presented solution would certainly not burden the companies’ budget considerably, as it would practically only replace the active parts of the forming equipment, which would speed up this work, make it more attractive, and would make it relatively easy for any just-trained worker and increase safety at work [17,18,19,20,21,22,23].

The process of magnetic forming is an idea that has been developed for several years. The authors applied the process of magnetic forming for the bending of metal sheets. To date, it is the bending of metal sheets specimens with smaller dimensions. The research has been tested for the forming of circumferential rings on the sheet metal extract. The efforts of the researcher have resulted in the performance of forming metal sheets in an open magnetic field, as well as experiments focusing on connecting pipes using a magnetic field [24,25,26,27]. The use of the magnetic phenomenon can also be found for the welding of metal materials by means of a magnetic impulse [28,29].

On the basis of the above overview, it can be stated that electromagnetic forming is one of the most advanced and environmentally friendly metal-forming technologies and awaits a great future in this century.

The main goals of this research are to present:

- The design and the construction of an experimental device intended to be used for the forming of metal sheets by a magnetic impulse as an unconventional way of forming metal sheets.
- The results of an experimental work conducted by means of the designed experimental device and to evaluate the obtained results in detail. Emphasis is especially placed on determining the achieved bending angle of experimental specimens.
- Novelty in the field of the forming of metal sheets by means of an ecological and economical way, namely, by means of the magnetic impulse to bend metal sheets with smaller dimensions.

The basis of the established hypothesis has been to investigate the possibilities of using a magnetic impulse for bending the metal sheet specimens. The analysis of the process has determined that it is necessary to design the forming equipment for bending the metal sheet specimens together with the design of the active parts of the forming device. The proposed bending angle of the metal sheets shows that it would be possible to create a bend with other values of angles, including the right angle, which is the most common bend angle in practice. It turns out that the entire process has to be analyzed from a theoretical point of view, as well as the application of relevant physical knowledge together with the necessary mathematical apparatus. The results obtained for the performed research are presented and described below, and they prove that the determined hypotheses are properly considered.

The entire area of the presented research was basically limited, so that the role of the application of the magnetic impulse applied for bending the metal sheet specimens stands out. This was only investigated in the area where the magnetic impulse can be used. Efforts were focused only on this area. In the case of an extension of processes, the entire researched area would grow considerably. The aim is, primarily, to verify the suitability of the magnetic impulse in the field of metal sheet forming, which clearly defines the field of our research.

## 2. Design and Construction of an Experimental Device

**Figure 1:**
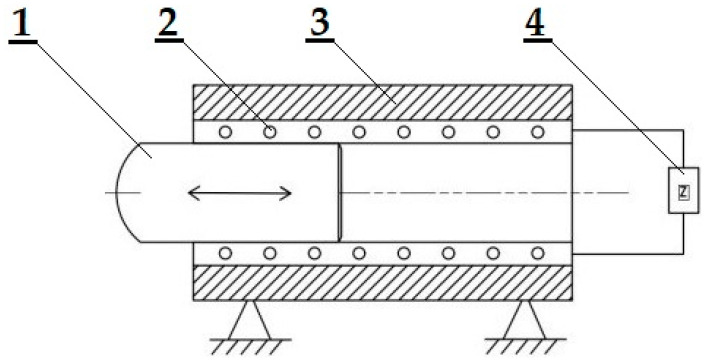
An application of the magnetic field with a free core: 1—a solenoid, 2—a coil, 3—a core, and 4—an electric circuit.

Figure 1 shows an idea of the construction and design of an experimental device for performing the experiments. Attention has been paid to the free core-forming, namely, for bending the metal sheet specimens. The experimental device was developed, designed, and manufactured by the researchers during their scientific activities.

The principle of core movement in a solenoid cavity comes from the physical principle. The magnetic field in the solenoid cavity is much stronger than around the solenoid. Lines of force cause the core inside the solenoid to move. The punch energy causes the core movement and it is generated by the capacitors in which the electric energy is accumulated. The kinetic energy is just the factor that effects the result of the forming of the specimens.

This work contains the following symbols that were used for the formulations:

- *F* [N]—a quantified force moving the solenoid core;
- *z* [-]—the number of coil treads;
- *I* [A]—the current intensity in the solenoid;
- *μ*_0_ [N∙A^−2^]—the permeability of air, *μ*_0_ = 4∙*π*∙10^−7^;
- *A* [m^2^]—a cross-sectional area of the solenoid core;
- *h* [m]—an air-gap width between the core and coil of the solenoid;
- *m* [kg]—the weight of the solenoid core;
- *g* [m∙s^−2^]—the gravitational acceleration, *g* = 9.81 m s^−2^;
- *A*_0_ [m^2^]—a core area with the diameter *d*_1_, *d*_1_ = 17 mm, *A*_0_ = 2.2698 × 10^−4^ m^2^;
- *A_s_*_1_ [m^2^]—a core area with the mean diameter of a cone *d*_2*s*_;
- *A*_1_ [m^2^]—a core area with the diameter d_3_;
- *l*_1*z*_ [m]—an active length of the core with a diameter *d*_1_ in the solenoid at the beginning of a test;
- *l*_2_ [m]—an active length of the core with a diameter *d*_2*s*_ in the solenoid during a test;
- *l*_3_ [m]—an active length of the core length with a diameter of d_3_ in the solenoid during a test;
- *l*_s_ [m]—an active length of the solenoid coil;
- *x* [m]—a displacement of the core from the zero position at the beginning of the test in the direction of magnetism;
- *h*_0_ [m]—the minimal width of a gap between the core and the coil of the solenoid;
- *h_s_*_1_ [m]—the mean width of a width of the cone between the core and the coil of the solenoid;
- *h*_1_ [m]—the maximal width between the core and the coil of the solenoid;
- *d*_2_ [m]—the average diameter of a cone part;
- *d*_2*M*_ [m]—the lower diameter of a cone part;
- *d*_3_ [m]—the diameter of the punch;
- *d*_1_ [m]—the diameter of the core;
- *F_g_* [N]—the gravitational force of the core;
- *F_N_* [N]—the normal force due to the gravitational force *F_g_* of the core;
- *F_T_* [N]—the tangential force due to the gravitational force *F_g_* of the core and the friction;
- *F_zp_* [N]—the resistance force of a specimen;
- *D* [m]—the internal diameter of the solenoid;
- CoG—the center of gravity of the core;
- *x_CoG_* [m]—the coordinate of the center of gravity of the core during its movement.

### 2.1. An Electrical Circuit for the Experimental Device

An electrical circuit is a supporting part of the experimental device, where a magnetic field (pulse) is generated, and which forms the experimental specimens (metal sheets) with the help of a forming tool.

Forming performed by means of the magnetic pulse (i.e., the force exerted by an application of the coil) can be divided into two main groups:

1. Forming with a free core: the core moves in the horizontal direction in the solenoid cavity and, when it impacts the experimental specimens, its movement is stopped;
2. A resilient core: the core is joined into a functional unit by means of a spring ensuring the displacement of the core after reaching the top dead center to the initial position.

The main purpose of the research was to verify the method of forming metal sheets by means of a magnetic impulse and to obtain relevant new knowledge and findings with the help of experiments, which will be generalized to applicable conclusions. The following mathematical description of this phenomenon was devoted to the movement of the free core in the solenoid cavity.

### 2.2. A Movement of the Core in the Solenoid Cavity

**Figure 2:**
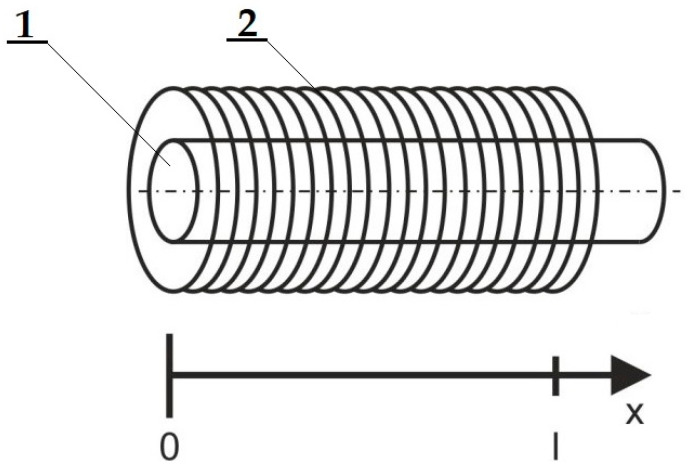
A scheme of a free core-motion in a solenoid: 1—a core, 2—a coil.

It is important to study the movement of the core in the solenoid cavity, as it directly depends on the fact that the active part coupled to the core will move. The force is the derivation of energy with respect to the position of the valve [30,31]. This requires the expression of the change of inductance with position *L*(*x*). The coordinate system can be defined as it is shown in Figure 2.

At *x* = 0, the anchor is completely inside the coil. At *x* = 1, the anchor is at the input edge of the coil. For these two positions, the inductance can be easily defined using the standard coil inductance approximation:

(1) $$L\left(0\right)=L_{0}=\frac{\mu_{r}\cdot \mu_{0}\cdot n^{2}\cdot S}{l},$$

(2) $$L\left(l\right)=L_{l}=\frac{L_{0}}{\mu_{r}}=\frac{\mu_{0}\cdot n^{2}\cdot S}{l}.$$

It may be advantageous to define *l* a little further outside the coil, so that the apparatus avoids the edge of the magnetic field *B*, thereby improving the accuracy. However, for simplicity, the spool length in (2) was used and an alternative way to produce this setting is provided below. *L*(0) was much larger than *L*(*l*), because the relative permeability of the iron fitting *μ_r_* was much larger than the permeability of free space *μ*_0_. *L*(*x*) differed monotonically between these two extremes, and the exact shape of this change in position depended on the design and shape of the solenoid. An accurate model of this variation requires the creation of a detailed spatial model (such as a finite element model) of a solenoid that is a magnetic field. A reasonable approximation for a cylindrical solenoid is needed for this purpose, and the exponential decomposition is:

(3) $$L\left(x\right)=L_{0}\cdot e^{-\frac{\alpha }{l}\cdot x}$$

If necessary, this value has the value *L*_0_ at *x* = 0 and the parameter α must be chosen so that $L\left(t\right)=\frac{L_{0}}{u}$.

An *α* value that is slightly less than this, but greater than unity, has the same effect as the movement of *l* and the outer coil:

(4) $$\alpha =\ln\left(\mu_{r}\right).$$

Hence, the force can be derived as:

(5) $$F\left(x\right)=\frac{V^{2}}{2\cdot R^{2}}\cdot \frac{\alpha }{l}\cdot L_{0}\cdot e^{-\frac{\alpha }{l}\cdot x},$$

(6) $$F\left(x\right)=\frac{I^{2}\cdot \mu_{r}\cdot \mu_{0}\cdot n^{2}\cdot S}{2\cdot l^{2}}\cdot \alpha \cdot e^{-\frac{\alpha }{l}\cdot x}.$$

The variables of the relations are: *S*—an area inside the solenoid (mm^2^), *n*—the number of solenoid coils, and *I*—current (A). 

The calculation is obtained from the research presented in [27]. The author presented $V=\frac{I}{R}$.

## 3. A Mathematical Model of the Device Operation with a Stamp Using Experimentally Acquired Data

**Figure 3:**
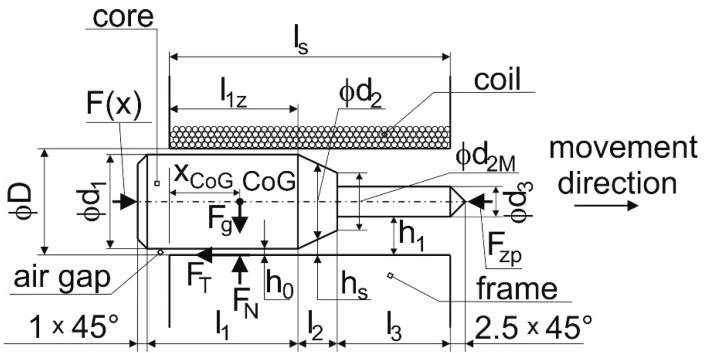
A schematic drawing of a solenoid cross-section with the geometry.

The used electromechanical solenoid HCNE1-1578 made by Zheijang Huaxin Electromechanical Co., Ltd. consists of an electromagnetic induction coil with *z* = 475 treads around a movable slug. The coil was shaped so that the armature can move inside it, thus altering the coil’s inductance and becoming an electromagnet. For the purposes of conducting the experiment for which the device was designed, the design of the solenoid punch was changed. A schematic drawing of the punch with its geometry is shown in Figure 3. Due to the design change, it was necessary to present calculations describing this change in order to determine their impact on the mathematical model of the punch’s behavior.

The main electric characteristics of the solenoid used is the current (Figure 3) defined by the equation:

(7) $$I\left(t\right)=-98,377\cdot t^{3}-4678.9\cdot t^{2}+218.54\cdot t+0.944$$

This current did not depend on the time interval of the changing current, with which the solenoid could be loaded. This is the reason why the current by which the solenoid was powered could not reach its maximum capacity. Instead, the current steadily increased until it was limited by the direct resistance of the solenoid. The inductor stored the energy in the form of a concentrated magnetic field.

**Figure 4:**
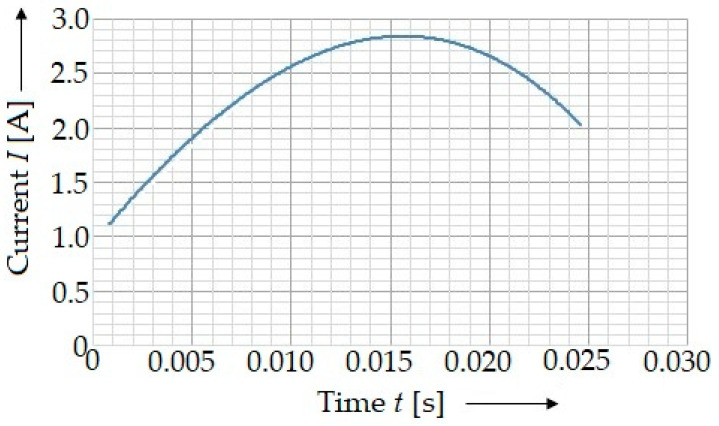
A course of the characteristics of the current in the solenoid determined experimentally by means of the ammeters.

As it can be observed in Figure 3, a core moves in a solenoid coil in the arrow direction (it is marked in Figure 3). In this view, the core moves from left to right. The right end of the core punches a metal sheet against a holder (Figure 4) and it results in the formation of a bend.

If there is still current in the conductor, a small magnetic field is created around the wire. The magnetic field becomes very concentrated with the solenoid wire wound around the coil. Such a design for an electromagnet can be used to control mechanical valves using an electrical signal. When the solenoid is under voltage, the current increases, causing the magnetic field to expand until it is strong enough to move the punch (armature), overcoming the friction force between the armature and coil, and the force from the acceleration of the punch’s linear movement. The movement of the punch contributes to increasing the concentration of the magnetic field, if the magnetic mass (the punch is made of structural steel) moves in the current direction from the magnetic field.

The value of the instantaneous force acting on the solenoid punch is calculated as follows:

(8) $$F={\left(z\cdot I\right)}^{2}\cdot \mu_{0}\cdot \left[\frac{S}{2\cdot h^{2}}\right],$$

where *F* [N] is a quantified force moving the solenoid punch, *z* [-], number of solenoid coils, *z* = 475 coils; *I* [A], the intensity of current passing through the solenoid, *I* = 0 ÷ 2.82 A; *μ*_0_ [N∙A^−2^], air permeability *μ*_0_ = 4∙*π*∙10^−7^; *S* [m^2^], cross-sectional area of the solenoid punch; and *h* [m], air-gap between the solenoid punch and coils.

As in Equation (8) above, there are a number of independent input variables influencing the instantaneous value of force *F*; they shall be reduced into a single dependent output variable. For the experiment, it was important to identify the punch velocity and acceleration; since we were looking for a mathematical model of the differential equation of motion describing the work of the device, all the variables were expressed as a function of trajectory *x* [m] that the punch performed in real time with the velocity and acceleration variables.

Equation (8) works correctly if the punch *S* [m^2^] cross-section size is a constant value. However, the change in the punch’s shape due to the structural modification resulted in the change in the instantaneous value *A*, which can be reduced to its real mean value dependent on the movement of *x*. Figure 3 shows the punch position at the beginning of the experiment. Determining the position of the center of gravity appeared to be important in order to identify the number of contact points at which the normal forces occurred between the punch and the coil. Since the punch center of gravity (determined both analytically and numerically using the Catia software (Dessault Systèmes, Vélizy-Villacoublay, France)) was located to the right of the tilting point *P*, only one normal reaction occurred. An attempt to move the punch against the coil produces a friction force acting against the movement of the punch, which results from the following condition:

(9) $$\sum_{i}F_{ix}=m\cdot a_{x}\Rightarrow m\cdot a_{x}=F-F_{T}.$$

The static equilibrium condition in the direction *y* produces:

(10) $$\sum_{i}F_{iy}=0\Rightarrow F_{g}-F_{N}=0.$$

For gravitation force *F_g_*, the following equation applies:

(11) $$F_{g}=m\cdot g,$$

where *m* [kg] is the weight of the solenoid punch after the structural modification, *m* = 0.1016 kg and *g* [m∙s^−2^], and is the value of the considered acceleration due to gravity, *g* = 9.81 m∙s^−2^.

Since the length of the punch was graduated and the space in which it moved was cylindrical, and since the punch material was considered isotropic, it was possible to determine the equivalent mean area of the punch *A_s_* in the position according to Figure 3; that is, at the beginning of each test, using the static moment of the area:

(12) $$S_{s}=\frac{S_{0}\cdot l_{1z}+S_{s1}\cdot l_{2}+S_{1}\cdot l_{3}}{l_{s}},$$

where *S*_0_ [m^2^] is the punch area with the diameter *d*_1_, *d*_1_ = 17 mm, *S*_0_ = 2.2698 × 10^−4^ m^2^; *S_s_*_1_ [m^2^] is the punch area with a mean cone diameter of *d*_2*s*_, *d*_2*s*_ = 12 mm, *S_s_*_1_ = 1.131 × 10^−4^ m^2^; *S*_1_ [m^2^] is the punch area with the diameter *d*_3_, *d*_3_ = 5 mm, *S*_1_ = 0.19635 × 10^−4^ m^2^; *l*_1*z*_ [m] is the active length of the punch with the diameter d_1_ in the solenoid at the beginning of the test, *l*1*z* = 24 mm; *l*_2_ [m] is the active length of the punch with the diameter *d*_2*s*_ in the solenoid during the test, *l*_2_ = 11 mm; *l*_3_ [m] is the active length of the punch with the diameter *d*_3_ in the solenoid during the test, *l*_3_ = 43 mm; and *l_s_* [m] is the active length of the solenoid coiling, *l_s_* = 78 mm.

To determine the precise mathematical model of the quantification of the effects that the solenoid had on the punch, Equation (12) was not sufficient due to the constantly varying size of the solenoid punch cross-section, which was different in each position *x*. A more accurate value can be obtained by the modification of Equation (12) using planimetry, where the current value of the cross-section is given by a linear change of parameter x:

(13) $$S_{\left(x\right)}=\frac{S_{0}\cdot \left(l_{1z}+x\right)+S_{s1}\cdot l_{2}+S_{1}\cdot \left(l_{3}-x\right)}{l_{s}},$$

where *x* [m] represents the movement of the punch from the zero position at the beginning of the test in the direction of magnetism.

**Figure 5:**
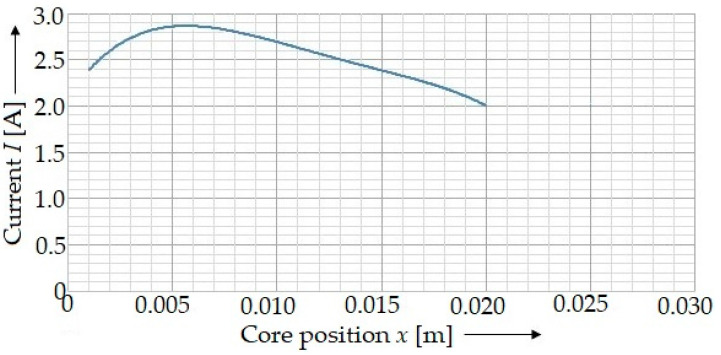
A calibration curve of the intensity of the current passing through a solenoid curve and a punch position *x*.

This causes a change in the instantaneous value of the punch cross-section in the calculation, and the mathematical model thus recalculates the value of the quantified force from the instantaneous punch area *S*_(*x*)_. A similar problem appeared in the case of the air-gap value between the punch and solenoid. It follows from Figure 5 that the value was different due to the punch graduation, and increased from the minimum value to the left (Figure 5) to the maximum presented to the right (Figure 5). Since the punch length was graduated and the space in which the punch moved was cylindrical, and the solenoid magnetic field was considered uniform and thus constant, it can be expressed by Equation (14) that, at the beginning of the test, the equivalent air gap can be calculated as follows:

(14) $$h_{s}=\frac{h_{0}\cdot l_{1z}+h_{s1}\cdot l_{2}+h_{1}\cdot l_{3}}{l_{s}},$$

where *h*_0_ [m] is the minimum gap between the punch and the coiling, *h*_0_ = 0.025 mm; *h_s_*_1_ [m] is the mean gap of the cone between the punch and the solenoid coiling, *h_s_*_1_ = 2.525 mm; and *h*_1_ [m] is the maximum gap between the punch and solenoid coiling, *h*_1_ = 6.025 mm.

To obtain a precise mathematical model for quantifying the solenoid-force effects on the punch, Equation (10) was not sufficient due to the constantly varying size of the gap between the solenoid punch and coiling, which was different for each position *x*. A more precise value can be obtained by modifying Equation (15) to the following equation:

(15) $$h_{\left(x\right)}=\frac{h_{0}\cdot \left(l_{1z}+x\right)+h_{s1}\cdot l_{2}+h_{1}\cdot \left(l_{3}-x\right)}{l_{s}},$$

**Figure 6:**
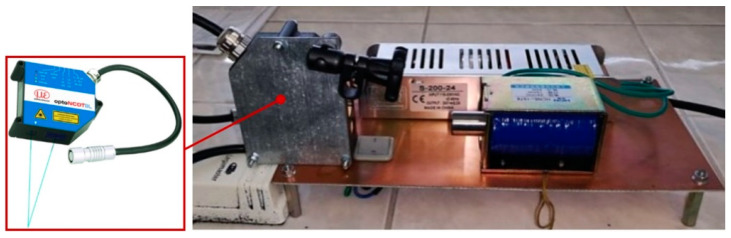
The determination of the acceleration of the solenoid punch by means of the analytical method (blue curve) and by means of the experiments (red curve).

By considering the experimentally determined current characteristics of the solenoid used (Figure 6), it can be found out that the value of the current was not constant, especially since the time in which the magnetic field acted on the punch was so brief (in the order of thousandths of seconds). The equation of the current dependence over time (Figure 4) defined by Equation (7) was a third order polynomial, which, however, explains the dependence of current on time. Due to the instantaneous quantification of the coil-force effect on the punch, depending on the position of the punch *x*, the calibration dependency of the solenoid current on position *x* should be created (Figure 5).

The equation of the current dependent on the trajectory *x* (Figure 5) is presented as follows:

(16) $$I_{x}=-5\times 10^{7}\cdot x^{4}+3\times 10^{6}\cdot x^{3}-47,642\cdot x^{2}+333.66\cdot x+2.0992.$$

The application of (13), (15), and (16) to Equation (8) results in the following:

(17) $$F={\left(z\cdot I_{\left(x\right)}\right)}^{2}\cdot \mu_{0}\cdot \left[\frac{S_{\left(x\right)}}{2\cdot h_{\left(x\right)}^{2}}\right],$$

(18) $$F={\left(z\cdot I\right)}^{2}\cdot \mu_{0}\cdot \left[\frac{\frac{S_{0}\cdot \left(l_{1z}+x\right)+S_{s1}\cdot l_{2}+S_{1}\cdot \left(l_{3}-x\right)}{l_{s}}}{2\cdot {\left(\frac{h_{0}\cdot l_{1z}+h_{s1}\cdot l_{2}+h_{1}\cdot l_{3}}{l_{s}}\right)}^{2}}\right],$$

(19) $$F={\left(z\cdot I_{\left(x\right)}\right)}^{2}\cdot \mu_{0}\cdot \left[\frac{\frac{S_{0}\cdot l_{1z}+S_{0}\cdot x+S_{s1}\cdot l_{3}-S_{1}\cdot x}{l_{s}}}{2\cdot {\left(\frac{h_{0}\cdot l_{1z}+h_{0}\cdot x+h_{s1}\cdot l_{2}+h_{1}\cdot l_{3}-h_{1}\cdot x}{l_{s}}\right)}^{2}}\right],$$

(20) $$F={\left(z\cdot I_{\left(x\right)}\right)}^{2}\cdot \mu_{0}\cdot \left[\frac{\frac{S_{0}\cdot l_{1z}+S_{s1}\cdot l_{2}+S_{1}\cdot l_{3}+x\cdot \left(S_{0}-S_{1}\right)}{l_{s}}}{2\cdot {\left(\frac{h_{0}\cdot l_{1z}+h_{s1}\cdot l_{2}+h_{1}\cdot l_{3}+x\cdot \left(h_{0}-h_{1}\right)}{l_{s}}\right)}^{2}}\right],$$

(21) $$F={\left(z\cdot I_{\left(x\right)}\right)}^{2}\cdot \mu_{0}\cdot \left[\frac{S_{s}+x\cdot \frac{\left(S_{0}-S_{1}\right)}{l_{s}}}{2\cdot {\left(h_{s}+x\cdot \frac{\left(h_{0}-h_{1}\right)}{l_{s}}\right)}^{2}}\right].$$

By applying the substitution for the constants, the result is as follows:

(22) $$c_{1}=\frac{\left(S_{0}-S_{1}\right)}{l_{s}},$$

(23) $$c_{s}=\frac{\left(h_{0}-h_{1}\right)}{l_{s}},$$

By substituting the relevant values in Equations (22) and (23) for the constants of the mutual structure of the punch and solenoid, *c*_1_ = 2.658 × 10^−3^ m and *c*_2_ = 0.077 (-) are determined. The equation is as follows:

(24) $$F={\left(z\cdot I_{\left(x\right)}\right)}^{2}\cdot \mu_{0}\cdot \left[\frac{S_{s}+x\cdot c_{1}}{2\cdot {\left(h_{s}-x\cdot c_{2}\right)}^{2}}\right].$$

From the analytically determined result of force *F*, the friction force *F_T_* = 0.1 N must be subtracted, and the result must be divided by the punch mass. This way, it is possible to obtain a punch acceleration value depending on the position *x*. The determined dependence (16) was processed into a clear graph (Figure 6).

It was possible to determine the equation for the solenoid punch acceleration by means of the analytical method using Excel (Microsoft Corporation, Redmond, WA, USA):

(25) $$a=-3\times 10^{9}\cdot x^{4}+2\times 10^{8}\cdot x^{3}-3\times 10^{6}\cdot x^{2}+30,197\cdot x+22.648.$$

**Figure 7:**
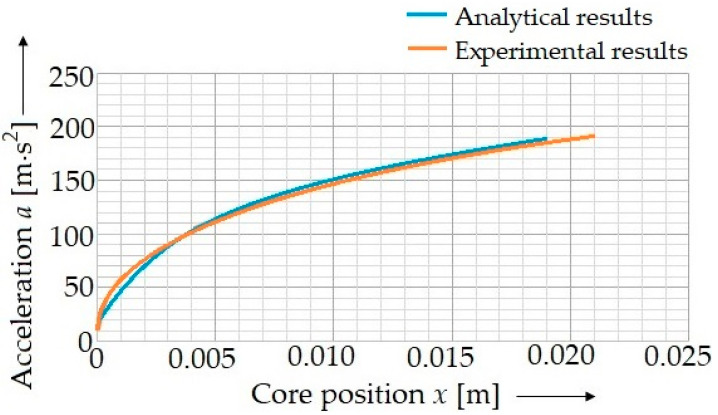
The determination of punch acceleration by means of experiments.

The acceleration value was determined experimentally using the ILD 1700 optical sensor (Micro-Epsilon Messtechnik Ltd., Ortenburg, Germany) (Figure 7). OptoNCDT1700 consists of a laser optical sensor and a signal conditioning electronic device. The sensor uses the principle of an optical triangulation, i.e., a visible modulated point of light is reflected on the target’s surface. The distance is linearized and delivered via the analog or digital interface. The measured values were exported into the Excel software, where the punch acceleration as the dependence on the traveled trajectory *x* was plotted (Figure 6). The maximum punch velocity was observed at the moment when the trajectory traveled a distance of *x* = 20 mm. At this moment, the instantaneous punch velocity was *v* = 2.334 m∙s^−1^.

Analytically, it is possible to write the motion equation as follows (26):

(26) $$\frac{d^{2}x}{dt^{2}}=\left\{{\left[z\cdot I_{\left(x\right)}\right]}^{2}\cdot \frac{\mu_{0}}{m}\cdot \left[\frac{S_{s}+x\cdot c_{1}}{2\cdot {\left(h_{s}-x\cdot c_{2}\right)}^{2}}\right]\right\}-g\cdot f.$$

## 4. Experimental Tests

**Figure 8:**
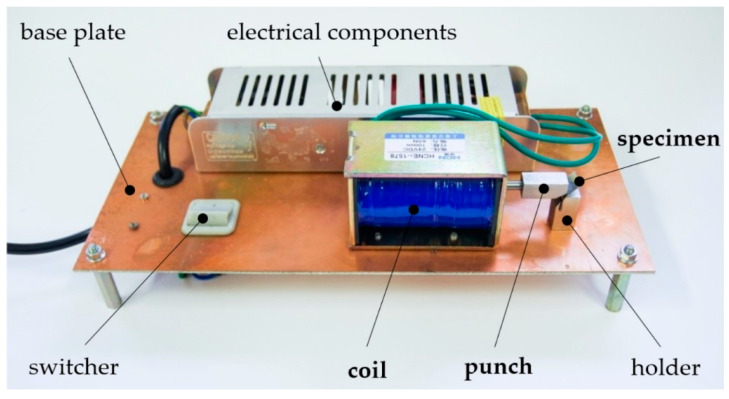
The equipment used for the experimental bending of sheet metal specimens by a magnetic pulse.

The experimental work was performed on the device itself, which is shown in Figure 8. As it can be observed, the experimental device consisted of several components. The main part was a base plate. Other components were mounted onto it. Electrical components included the needed part of the electrical circuit, for which a detailed description is not the subject of the current research. A switcher served to activate the device. A holder kept the metal sheet required for the bending position. It also determined the requested angle of the metal sheet. The most important components marked in Figure 8 are written in bold and they are, namely, a coil, a punch, and a specimen. The magnetic pulse was generated in the coil. Its effect then moved the punch against the specimen. The punch movement was stopped by a hit to the holder together with the specimen.

Forming was applied with the free core, where metal sheet specimens were bent to an angle of 30°. Sheet metal clippings 0.15 mm and 0.20 mm thick were used as specimens. Seven specimens were formed for each thickness. The chemical composition of the specimens is shown in Table 1.

**Figure 9:**
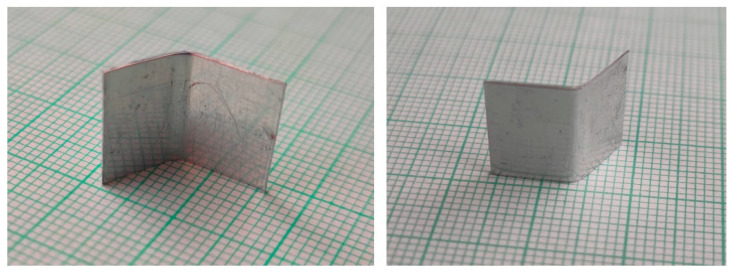
The metal sheet specimens after experimental tests.

The specimens for the experiments were sheet metal strips cut into a rectangular shape with the dimensions of 23 mm (length) and 12 mm (width). The strips had two different thicknesses, namely, 0.15 mm and 0.20 mm. Figure 9 shows the final shape after the forming process. The specimen is depicted from both sides.

**Figure 10:**
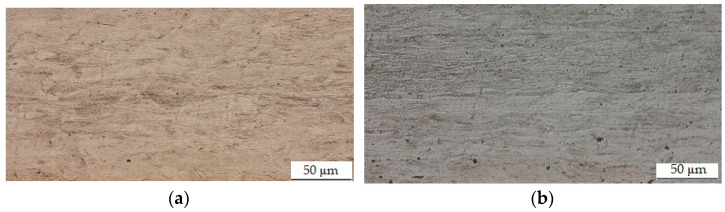
(**a**) The metallography of the sheet metal with the thickness of 0.15 mm; (**b**) the metallography of the sheet metal with the thickness of 0.20 mm.

Figure 10 shows the metallography of the material used in the experiment. The surface is etched by Nital 2%.

## 5. Results and Discussion

**Figure 11:**
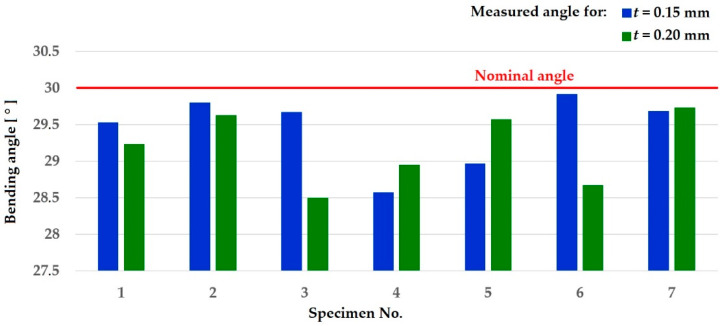
The results of the bending angle of specimens with the thicknesses of 0.15 mm and 0.20 mm and a comparison to the nominal set angle (defined by the holder).

The performed experimental work revealed several important findings. Two kinds of material with thicknesses of 0.15 mm and 0.20 mm were formed using the application of the magnetic pulse. It was measured on a GAM 270 MFL instrument (Robert Bosch Power Tools Ltd., Leinfelden-Echterdingen, Germany). After switching on the magnetic circuit, a magnetic pulse was generated, which subsequently formed the material into an angle, which should occur according to the fixed part and the 30° bend. The results in Table 2, together with the graph in Figure 11, show the parameters of the bent specimens after the experiments. If we obtain the results of the specimens with a thickness of 0.15 mm at a bending angle of 30°, then the maximum value of the deviation from the required bending angle is 1°26′ and the minimum is 5′. The bending process can be evaluated as a stable process during the experiments. The other specimen type with the thickness of 0.20 mm was also bent to the angle of 30°. The results at a bending angle of 30° are the maximum value of the deviation 1°30′ and the minimum 12′. The bending process is also stable, as in the case of forming the specimen with the thickness of 0.15 mm.

The results obtained from the experiments with the application of a free core (punch) by a magnetic pulse show that such a shaping method is suitable for several reasons. The first is the speed of the process, when the work itself is a period of time expressed in hundredths of seconds. The second advantage is from the point of view of easy automation when using this type of forming. The analysis of the results from Table 2 shows that this is a relatively stable process, as the differences between the maximum and minimum values are minor.

The research presented in [19,20] presents the bending of sheet metal specimens in a magnetic field. The results show that the differences in magnetic pulse bending are minimal in comparison to the method described there. Forming in the field of magnetic phenomena is possible and it proves the productivity as well as feasibility of bend creations. The metal sheet specimens suitably served as an experimental material for the research activity and, based on the results and findings, it can be concluded that the solution is suitable for industrial use (of course, when using more powerful devices). It is also important to mention the ecological factor of the process. The necessary electricity, which powers the equipment, can also be generated by means of alternative sources and the workplace does not burden the environment as in the case of using a conventional forming machine and all-metal forming tools.

During the research of this method, it was found that the indicated and tested method supported by the analytical analysis of the issue proved its suitability for further development. It is highly desirable to use knowledge from the field of physics, which can reveal new perspectives concerning the processes of metal sheet forming with the application of non-traditional (unconventional) methods. The method proved suitable for its application in the industry, where simple shapes are produced, e.g., bends. It turned out that it was necessary not only to follow the established direction of the research, but also to extend it to other related implementation processes, in this case, metal sheet parts.

## 6. Conclusions

The main objective of the presented research focused on the use of magnetic phenomena in metal-forming technology. The goals of the current work were achieved and met. An original device was developed and constructed, which ensured that the experiments with sheet metal bending were performed. The laboratory experimental solution showed the way in which physical knowledge can be conveniently applied to industrial production. It was confirmed that a relatively simple solution with the application of a magnetic field ensures the accurate, fast, and high-quality bending of metallic materials. 

The achieved goals of the presented research can be concluded as follows:

1. Design of the original experimental-forming equipment;
2. Verification of the suitability of the equipment in experimental conditions;
3. Obtaining information about the course of the bending process and the results of the experiments.

The practical contribution of the research is that the device used to form metal sheet components by a magnetic impulse was successfully tested. It refers to the proper direction of this research. The experiments confirm that this field represents a new domain, which is exclusively based on physical knowledge. They are actively transformed to a practical solution. The useful contribution of this study is a design of the device, a calculation model of the parameters, and the summarized results of the performed experiments. Future research needs to verify how a modified solution could be used for volume forming, i.e., for forming cavities of forming tools. Cavities can be created by a magnetic impulse alone. A significant contribution to the practice is the fact that the solution is more environmentally friendly than conventional solutions, which require large forming machines and considerable amounts of energy. The electric energy applied to the mentioned solution can be obtained from alternative sources, which is one of the means of achieving sustainable development and a reduction in the burden on the natural environment.

Another novelty of the experimental research is that it brings the use of scientific methods into practice, especially in the production of bends of metal sheet parts. By using a simple device, essentially a solenoid with a movable core that moves in its cavity and the action of a magnetic shock, it ensures the formation of the required angle on the metal sheet sample. The main goal is to compensate the movement of the slider of the hydraulic press by the impact of the solenoid core, onto which the active part of the forming device is mounted. Moreover, the novelty is also recognized in terms of a greater greening of the process, in the sense that it does not require a forming machine, for example, in the case of using hydraulic fluid, which can be a source of environmental burden in the event of an accident and also protects the worker from injury.

The results obtained also confirm the suitability of the mentioned solution for applications in the production process. Creating bends is one of the most common jobs in the production of sheet metal parts. The implementation of this type of work combines several advantages: speed of production, accuracy, and very good final quality of bends. The reproducibility of the results is also high. The described solution complements and expands the sphere of sheet metal forming. The published results provide a good basis for further research in this area.

The final conclusions of the findings and novelties of the research are:

1. The new experimental device for forming metal sheets was designed and manufactured.
2. The working principle of the experimental device was described by the mathematical apparatus.
3. The functionality of the experimental device was verified by experiments.
4. The characteristics of the experimental device (focusing on the acceleration of the solenoid core) were determined by experimental tests and calculated by means of the mathematical apparatus. The results a high agreement with each other.
5. The experimental device was used for the experiments to form metal sheets with the thicknesses of 0.15 mm and 0.20 mm. The results prove that the experimental device can be used for the bending of metal sheets.
6. The proposed method can be applied to the practice of bending metal sheets in case of the construction of the device with the needed parameters.
7. The forming of metal sheets can to contribute to the ecological and effective processes of forming metal sheets.

## Figures and Tables

**Table 1:** Chemical composition of the specimens’ materials (wt. %).

**Element**	***Si***	***Mn***	***S***	***Cr***	***Mo***	***Ni***
Specimen No. 1, *t* = 0.15 mm	0.94	1.29	0.026	17.0	0.52	6.57
Specimen No. 2, *t* = 0.20 mm	0.96	1.30	0.034	17.2	0.44	6.75
**Element**	***Cu***	***Nb***	***V***	***Pb***	***Fe***	
Specimen No. 1, *t* = 0.15 mm	0.34	0.01	0.06	0.02	73.7	
Specimen No. 2, *t* = 0.20 mm	0.21	0.016	0.10	0.029	72.9	

**Table 2:** Results of thickness experiments for specimens with the thickness of 0.20 mm.

Specimen No.	Bending Angle (°)		Deviation from the Required Angle (°)	
	*t* = 0.15 mm	*t* = 0.20 mm	*t* = 0.15 mm	*t* = 0.20 mm
1	29°32′	29°14′	28′	46′
2	29°48′	29°38′	12′	12′
3	29°40′	28°30′	20′	1°30′
4	28°34′	28°57′	1°26′	1°3′
5	28°58′	2934′	1°2′	26′
6	29°55′	28°40′	5′	1°20′
7	29°41′	29°44′	19′	16′

## Data Availability

Not applicable.
